# On the Uses of Turpentine

**Published:** 1854-01

**Authors:** 


### THE NORTH-WESTERN MEDICAL AND SURGICAL JOURNAL.

NEW SERIES.

VOL. III. JANUARY, 1854. NO. 1.

### ORIGINAL COMMUNICATIONS.

*Transactions of the Illinois State Medical Society, for the year* 1853. "On the Uses of Turpentine; by Samuel Thompson, of Albion, Illinois.

*A Dictionary of Practical Medicine*, Comprising General Pathology, the Nature and Treatment of Diseases, Morbid Strictures, &c: by James Copland, M. D., F. R. S. Re-published by Harper & Brothers, New York.

"RENDER UNTO CÆSAR THE THINGS THAT ARE CÆSAR S."

It is probably the first time that these words have been adopted as the motto of a medical critic; they were used by a remarkable person, on a remarkable occasion, and contain a sentiment that ought to influence the heart and conduct of every physician. But, we write it with sorrow, medical literature, and especially medical periodical literature, affords too many examples where the exact superscription of Cæsar has been retained and his image effaced—and more still, where the ideas and practice of one physician have been copied and published by another, without any allusion to the source whence they were borrowed: and in many instances of this dishonorable conduct, ignorance of prior publication would, in our opinion, be less justifiable than openly-confessed plagiarism. No one will deny the truth of these remarks—that they are appropriate, we think, will plainly appear in the sequel. We will briefly state that the preceding remarks are intended to apply generally to the authorities quoted by Dr. Thompson, and not to the Dr. himself, and that we are amongst those who have been benefitted by the doctor's observations at previous meetings of the State Society, and lamented his absence from the last; we admire his energy, professional honor and zeal, and had placed a higher estimate on his medical information than the present article justifies.

The Dr., after enumerating several cases in which he had prescribed turpentine, proceeds: "The first of these recorded cases in which I have used it, bears date 24th July, 1851, and, though I do not now remember upon whose suggestion I was first led to use it in the bold way I have since done, I suspect it was the paper of Dr. Hughes Willshire, in the 12th number of Braithwaite's Retrospect, on its uses in purpura, and wherein he mentions Dr. Copland's view of its modus operandi, to be absorbed into the blood, (Query, the turpentine or Copland's view,) and its thence producing an astringent effect upon the capillary vessels." Dr. Willshire has no article in that No. of the Retrospect, but Dr. Neligan has one on Purpura, in which turpentine in full doses is used and recommended as a remedy in that disease, without any reference to Dr. Copland or his opinion. Nor is Dr. Thompson's description of Copland's view of its modus operandi exactly correct: 'There is much misapprehension as to the operation of full doses of turpentine, given either by the mouth or in enemata; many supposing that they increase vascular action in the brain."

"The reader will perceive, upon perusing the account (published in the *London Med. and Phys. Journal* for May and July, 1821) of the experiments I performed; 1st upon myself; 2ndly on the lower animals, and 3dly in numerous cases of disease—that this substance, given so as to act upon the bowels, either from the largeness of the dose, or by the aid of a purgative conjoined with it, is a powerful derivative from the brain, diminishes vascular action in serous membranes, and *restores lost tone* to the extreme capillaries, especially in exhaling surfaces. The extensive experience I have since had of this medicine, has confirmed these inferences, but has shown that it may be injurious in the hands of those who are not well acquainted with the exact circumstances in which it may be given with advantage."—*Copland's Dictionary*, article Erysipelas, published 1834.

"The chief hindrances to the employment of this substance (Turpentine) are, 1st. A mistaken view of the nature and consequences of its operation; 2d. Its nauseous or unpleasant effects; and 3d. The opinion that it cannot be retained by the stomach when nausea and vomiting are complained of. As to the *first* of these, I can assert that it is, according to the mode of its exhibition, antiphlogistic in acute inflammations, and more efficacious in arresting the progress and consequences of asthenic or diffusive inflammations than any other substance; while it possesses the property of accommodating, by its tonic and astringent operation, the vascular and capillary system to the state and amount of its contents, of lowering the frequency of the pulse, and of restraining effusion from serous and mucous surfaces. That it is unpleasant, and that it is sometimes thrown of the stomach, I admit; but in many such cases it is beneficial nevertheless, its emetic action, independently of the impression produced by it on a vital organ, occasionally being of service, and even actually required. In those cases where the irritability off the stomach is even the greatest, it not only is the most easily retained, but is actually the most efficient remedy for the removal of the irritability, which, in the opinion of many, is the chief reason against a recourse to it. But the exhibition of it by the month is often not the only, and sometimes not the most beneficial way of prescribing it; for it may also be administered in enemata, or applied externally, and occasionally, according to the nature of the case, even more efficaciously than in any other mode."–*Copland.* Art. Puerperal Diseases, published in 1850.

We have here given the first and the last of Dr. C.'s recorded opinion of the effects of full doses of Turpentine in our possession; and the reader by carefully remembering it will be able to see, as we proceed with this review, how applicable it is in many cases apparently of a very opposite character, but "involving similar functional or structural derangements."

Dr. Wood (*Practice of Medicine*, Vol. 1, Page 328) says, "It (turpentine) acts in some measure as a stimulant, but chiefly, I believe, as an alterative to the ulcerated surfaces in the intestinal mucous membrane."

Dr. Smith (*American Journal of Medical Sciences*, July 1850, page 168) remarks, "Turpentine, when taken internally, exerts a peculiar action on the mucous surfaces." After a careful perusal and re-perusal of his more lengthy article in the same journal, page 196, we cannot see that the Dr. has thrown any light, at least any *new light* on the modus operandi of this medicine—he speaks highly of it as a *counter-irritant*, *vermifuge*, *purgative*, *astringent*, and *styptic*—gives an extensive list of diseases in which it has been beneficially employed and in a note records the names of the Physicians who prescribed it. "It would be a useless task (he says) to cite all the cases and all the maladies, in which the admirers of this drug have found it advantageous. Suffice it to say, that in every instance where prejudice has not interfered, and where *ignorance has not prescribed*, this drug has obtained favor and proved itself a faithful friend." Our experience vouches for the truth of these remarks. The article of Willshire referred to by Dr. Thompson is not in our possession, and it would be useless to quote from Pereira as his work is in the library of every Physician who ought to practice medicine.

Dr. Thompson has given a list of diseases, which might have been considerably extended, in which Turpentine has been recommended and administered; and a reference to the systematic and periodical works in which the recommendations may be found. We now propose to take the diseases *seriatim*, and to show that Dr. Copland had previously used the drug in almost every instance; and that he has more clearly and fully stated, than any previous or subsequent writer, the exact pathological condition in each disease requiring the exhibition and use of Turpentine. This will be to us a pleasant task—for the Dr. has long been the "man of our counsel, a lamp unto our feet, and a light unto our path," safely leading us through many intricate and perplexing scenes of professional difficulty. And we trust that many of our readers will be profited by a clear exposition of the circumstances demanding the use of a remedy that, in our hands, has not been inferior to any in saving the human fabric from destruction.

If time and space permit—we shall close our remarks by a record of cases in which Turpentine has been successfully employed in all but hopeless conditions of disease; and we here venture to assert that if there be a medicine which will fortifiy the system against an attack of typhoid fever—*that medicine is Turpentine.*

Our experience of it as a prophylactic is too limited to speak positimely of its efficacy; and yet it is sufficiently extensive to encourage a hope that many cases of this fever may be prevented by a timely and persevering use of it by the healthy, when once the disease gets foothold in a family.

It is of no real practical advantage to the junior practitioner to be told that this or that remedy is recommended in this or that disease, unless at the same time it is plainly shown at what stage of the disease the medicine is required, and what are the peculiar features of the disease in that stage demanding its use; and also, when it is understood, the way in which it acts in curing the disease—or, when it cannot effect this—in arresting its further progress. Now should any of our readers meet with a case of iritis (we have not seen one for over twenty years) and, in consequence of seeing turpentine alluded to as a remedy in that disease, should depend upon it as his main or only agent, he will, most assuredly be wofully disappointed. It was to procure the absorption of effused lymph and prevent the iris adhering to contiguous tissues that Carmichæl used turpentine as a substitute for mercury, and he has precedence in this matter; his work on the use of turpentine in deep-seated inflammation of the eye was published in 1829. But this disease is so rare, that we will pass on to the typhoid pneumonia. Dr. Thompson alludes to one case in Braithwaite's Retrospect for 1840. "Dr. Huss considers the employments of the etherial oil of turpentine to be one of the greatest improvements in the treatment of certain complications of typhoid fever. It is of essential value, he says, in the typhoid form of pneumonia, which is so apt to show itself between the eleventh and the fourteenth day of the disease; particularly in those cases where the debility of the heart's action is announced by the absence or diminution of the first sound of that organ. The weakened heart is then unable to propel the blood through the weakened capillaries of the lungs, congestion ensues, and proceeds to inflammation of a low typhoid kind. The other indications for the employment of turpentine, in this complication, are cough, with viscid and more or less bloody expectoration, dulness on percussion over some portion of the chest, particularly at the back part, with crepitation and tubular or bronchial respiration there." He gave it every hour in small doses.—*British and Foreign Medical Review*, Oct., 1846, page 473.

"*Embrocations* with *spirits of turpentine*, applied over the chest or between the shoulders, are the most valuable remedies that can be used in this form of the disease, and in the advanced stages of the sthenic variety. The best mode of resorting to them is by means of two or three folds of flannel, of sufficient width to cover the greater part of the chest. These should be wrung as dry as possible out of hot water, be instantly sprinkled freely with the spirits of turpentine, and applied to the surface; taking care to cover them, when thus placed on the thorax, with a napkin, oil-skin, or other material which may prevent or much impede evaporation. This embrocation should be kept applied as long as the patient will endure it, or renewed from time to time. Instead of the spirits of turpentine, an embrocation consisting of equal parts of the compound camphor liniment, and of the turpentine liniment, with a little cajeput oil, may, after having been well shaken, be sprinkled on the warm flannel, and applied as thus directed. I believe that the inhalation of the vapor from this embrocation is partly influential in producing the benefit which accrues from it, and which I have witnessed in many cases."—*Copland's Dictionary*, vol. 2nd, page 777: London edition, and published in 1838. Article, Asthenic Inflammation of the Lungs.

We vouch for the utility of both modes, and have occasionally conjoined them; they are worthy of the attention of every physician who may be called upon to treat this frequently fatal form of Pneumonia. We recollect, some ten years ago, being called to a case of this disease that was considered hopeless by the friends and by the pretender who had been treating him. One tea-spoonful of turpentine was given every three hours, and hot flannels, moistened with turpentine, were applied to the chest, and covered with a piece of oiled newspaper, (our common covering). Occasionally the sheet was thrown over the face, so that the vapor should be thoroughly inhaled; in twelve hours the change for the better was very perceptible and the patient recovered. The pseudo-physician stood by and observed the whole proceeding, and treasured up the supposed information for future use. Some two weeks afterwards he used the remedy in both ways in a case of sthenic pneumonia, with the effect, however, of all but producing suffocation, and aggravating the patient's sufferings ten-fold. We saw him the next day, but he ultimately died. Truly does Dr. Copland observe, "that it may be injurious in the hands of those who are not well acquainted with the exact circumstances in which it may be given with advantage." The hyperæmia of sthenic, and the passive congestion of asthenic inflammation, are widely different pathological conditions, especially when the latter is associated with a truly typhoid state of the system. The state of vital power and vascular action is very dissimilar, and unfortunate, indeed, is it for the patient when the physician does not know it.

(*To be continued.*)

